# Broadening the definition of a bioinformatician

**DOI:** 10.3389/fgene.2015.00258

**Published:** 2015-08-04

**Authors:** David Roy Smith

**Affiliations:** Department of Biology, University of Western OntarioLondon, ON, Canada

**Keywords:** bioinformatician, bioinformatics, biologist, data scientist, informatician

I am currently an assistant professor in the biology department at a research-intensive university. When I interviewed for this job 3 years ago, it was loosely advertised as a bioinformatics position. At the time, I was studying genome evolution of eukaryotic algae—a topic that I am still actively engaged in Smith (2015). On a given day, I spend much of my research time staring at nucleotide sequences on a computer screen and theorizing about the evolution of genomes; thus, I feel comfortable calling myself a bioinformatician, or at the very least a scientist who primarily uses bioinformatics for his research. If asked, most of my colleagues, mentors, and students would also define me as a bioinformatician. But there is one small catch: I don't know how to program computer software or curate databases, and I am even quite pathetic at writing UNIX commands, which according to some precludes me from having the title of bioinformatician.

I imagine that many of the scientists reading this essay will consider me an imposter, an amateur who points, clicks, and stumbles his way through the complicated landscape of bioinformatics. But no matter what people may think, I won't be returning my bioinformatician badge anytime soon. As outlined below, I believe that as a research community we need to broaden our definition of what it means to be a bioinformatician, not restricting it to only those who develop software or design and maintain data resources. Specifically, I argue that the term bioinformatician should encompass the countless and ever growing number of scientists who use computers and bioinformatics programs to address fundamental questions in biology—from the origins of eukaryotic life (Burki, 2014) to the roots of genomic architecture (Smith and Keeling, 2015) to the evolution of malaria (Preston et al., 2014)—even if those scientists are not expert programmers themselves.

A recent article published in this journal took the opposite stance. Vincent and Charette (2015) explored the question “Who qualifies to be a bioinformatician?” and proposed that the title should be reserved to experts in the field of bioinformatics, which in their view means those “who understand the underlying mechanics of bioinformatics” and “conduct research based on a bioinformatics approach.” The authors further argued that bioinformaticians fall into two categories: those who work directly on bioinformatics algorithms and tools and those who architecturally design and maintain data resources. Their strongest and most contentious point, however, was that a “biologist who only uses bioinformatics tools to perform analyses but does not contribute [to] the conception of such tools …is not a bioinformatician.” Based on this definition, I—and many other researchers like me—do not qualify to be a bioinformatician, which makes me wonder: what, then, do you call a scientist who spends most of his or her day employing, but not developing, bioinformatics tools? Vincent and Charette (2015) suggested that a “strict user of bioinformatics tools could be an expert in another field; for example, a genomicist can use bioinformatics tools without being a bioinformatician.” But the words genomicist or phylogeneticist or transcriptomicist have a much more limited scope than the term bioinformatician, potentially limiting the job prospects and research opportunities of scientists who label themselves as such, and neither of these restrictive words likely encompass their broad skillsets.

One argument for a narrow interpretation of a bioinformatician is that it allows universities, human resources departments, and governing bodies to more accurately teach, recognize, and certify their respective students, employees, and members as bioinformatics “experts” (Vincent and Charette, 2015). The problem with this approach, in my opinion, is that it could discourage students and faculty from learning and engaging in bioinformatics research. For example, many of the one thousand second-year genetics students I taught this past semester would happily take a course on user-friendly bioinformatics software, but they may think twice about taking such a course if they thought that it wouldn't be considered “true” bioinformatics or that they could be labeled as pseudo-bioinformaticians. A number of these same students, however, would be immediately turned off at the thought of studying programming languages. Similarly, graduate students, postdocs, and faculty whose research revolves around bioinformatics tools but who cannot write scripts and are therefore branded “non-bioinformaticians” might be discouraged from applying for grants, awards, or jobs related to bioinformatics, even if they are suitable for the funding or position.

We should be striving to make the field of bioinformatics more inclusive rather than exclusive. Being elitist about who can call themselves a bioinformatician works against this goal. At various times in my research career, I have been ridiculed for not being able to develop bioinformatics programs or write PERL scripts, even though my research productivity and creativity were on par with those doing the ridiculing. No one, however, has ever belittled me for not knowing how to build a thermal cycler, electron microscope, or sequencing machine; moreover, the inability to construct any of these devices does not preclude people from being molecular biologists. Likewise, not knowing how to design a genome assembly program or phylogenetics algorithm should not stop people from calling themselves bioinformaticians. By all accounts, Steve Jobs was a terrible programmer, but few would argue that he was a computer pioneer with an amazing knack for understanding technical concepts. Craig Venter is celebrated in the fields of synthetic biology and genome sequencing—fields where computer coding and biology blend together—yet as far as I know Ventor is neither an expert programmer nor a software developer.

I am not alone in thinking this way. Indeed, the paper by Vincent and Charette (2015) sparked significant online debate within the bioinformatics social media network, and based on Altmetric is among the most highly discussed articles currently published in the journal *Frontiers in Genetics*. Much of the discussion revolved around a metaphor that the authors used: the idea that just using bioinformatics software does not make you a bioinformatician, the authors argue, “is a little like saying that driving your car to work does not make you a mechanic.” Mick Watson, who is head of bioinformatics at Edinburgh Genomics, picked up on this in his blog Opiniomics, writing: “So which type of bioinformatician are you? Engineer, designer, builder, fixer (mechanic), or user? Oh wait, I forgot, the “user” isn't a bioinformatician. So what are they? Hello ‘Data Scientist’!!”.

Simon Cockell, unit manager at Newcastle University Bioinformatics Support Unit, also made insightful comments on his blog (called Simon's Blog) regarding the Vincent and Charette (2015) article. “There are no hard and fast rules about what a bioinformatician is and isn't. The label will mean different things to different people. But what it does involve is an unusually wide skillset, usually hard-won over many years, and the knowledge of when and where to apply those skills. It definitively doesn't involve looking down on hardworking practitioners in the field purely because they don't fit your elitist mold—the only thing this is likely to do is exclude those interested in the field…”

However, there is no denying that bioinformaticians often need to creatively assemble or combine bioinformatics tools into novel architectures or “pipelines.” These kinds of tasks do not necessarily require programing skills, but they do require a general understanding of the tools in the pipeline as well as the underlying theory and programming used to develop those tools (Loman and Watson, 2013).

In the near future, there will be even more sophisticated bioinformatics programs. In turn, bioinformatics (and bioinformaticians) will play an increasingly central and important role in science, medicine, and education, and might soon blend into our everyday lives (Oshlack, 2013; Chang, 2015)—for example, it is not hard to envision bioinformatics software running on smart watches. As more and more people come into contact with and become interested in bioinformatics, we will need a broader and broader range of people with diverse and differing abilities to design, develop, promote, teach, and carryout bioinformatics. The people involved in this work may not all fit a single definition of bioinformatician but should still be allowed fall under its umbrella.

Vincent and Charette (2015) make some excellent and compelling points in their article, and although I disagree with some of them, one of their final points resonated with me: “A good definition of a bioinformatician should not be based on a single concept …real bioinformaticians share a number of common characteristics …none of which [are] essential.” Perhaps a common characteristics that we share as bioinformaticians (and maybe this one should be essential) is a passion for using computers to understand the bewildering biological world that surrounds and encompasses us.

Defining the qualities of a bioinformatician is a challenging and incendiary topic, and one that was exemplified by two referees who reviewed an earlier version of this manuscript. One referee wrote, “I concur whole heartedly with the author. I've been analyzing other people's data for the last 20 years and have not written a single [line] of analytical software script.” The other referee wrote, “I disagree with the author. …While different students in different curricula have different needs, somebody getting a degree in bioinformatics should be adept at programming and comfortable operating in a Unix environment—that's my ‘line in the sand’ when I hire a bioinformatician for the core facility that I run.”

The latter referee concluded by saying: “In the end, one of the original merits of Vincent and Charette (2015) was to advocate for a ‘solid’ approach to bioinformatics skills development. Such an approach could help young scientists avoid disappointment later in their careers.” This is a salient point, and one that we should all keep in mind when we consider what it means to be a bioinformatician.

## Conflict of interest statement

The author declares that the research was conducted in the absence of any commercial or financial relationships that could be construed as a potential conflict of interest.
